# Leptin-induced ER-α-positive breast cancer cell viability and migration is mediated by suppressing CCN5-signaling via activating JAK/AKT/STAT-pathway

**DOI:** 10.1186/s12885-018-3993-6

**Published:** 2018-01-25

**Authors:** Inamul Haque, Arnab Ghosh, Seth Acup, Snigdha Banerjee, Kakali Dhar, Amitabha Ray, Sandipto Sarkar, Suman Kambhampati, Sushanta K. Banerjee

**Affiliations:** 1 Cancer Research Unit, VA Medical Center, Kansas City, MO USA; 2 Department of Medicine, University of Kansas Medical Center, Kansas City, KS USA; 3 Department of Anatomy and Cell Biology, University of Kansas Medical Center, Kansas City, KS USA; 4 Department of Pathology, University of Kansas Medical Center, Kansas City, KS USA; 5 Present Address: Syngene International Ltd, Clinical Development, Tower 1, Semicon Park, Phase II, Electronics City, Hosur Road, Bangalore, Karnataka 560100 India; 6 Present Address: Saint James School of Medicine, Anguilla, British West Indies USA; 7 Cancer Research Unit, Research Division 151, VA Medical Center, 4801 Linwood Boulevard, Kansas City, MO 64128 USA

**Keywords:** Leptin, CCN5, Breast cancer, Proliferation, Invasion and migration

## Abstract

**Background:**

In menopausal women, one of the critical risk factors for breast cancer is obesity/adiposity. It is evident from various studies that leptin, a 16 kDa protein hormone overproduced in obese people, plays the critical role in neovascularization and tumorigenesis in breast and other organs. However, the mechanisms by which obesity influences the breast carcinogenesis remained unclear. In this study, by analyzing different estrogen receptor-α (ER-α)-positive and ER-α-negative BC cell lines, we defined the role of CCN5 in the leptin-mediated regulation of growth and invasive capacity.

**Methods:**

We analyzed the effect of leptin on cell viability of ER-α-positive MCF-7 and ZR-75-1 cell lines and ER-α-negative MDA-MB-231 cell line. Additionally, we also determined the effect of leptin on the epithelial-mesenchymal transition (EMT) bio-markers, in vitro invasion and sphere-formation of MCF-7 and ZR-75-1 cell lines. To understand the mechanism, we determined the impact of leptin on CCN5 expression and the functional role of CCN5 in these cells by the treatment of human recombinant CCN5 protein(hrCCN5). Moreover, we also determined the role of JAK-STAT and AKT in the regulation of leptin-induced suppression of CCN5 in BC cells.

**Results:**

Present studies demonstrate that leptin can induce cell viability, EMT, sphere-forming ability and migration of MCF-7 and ZR-75-1 cell lines. Furthermore, these studies found that leptin suppresses the expression of CCN5 at the transcriptional level. Although the CCN5 suppression has no impact on the constitutive proliferation of MCF-7 and ZR-75-1 cells, it is critical for leptin-induced viability and necessary for EMT, induction of in vitro migration and sphere formation, as the hrCCN5 treatment significantly inhibits the leptin-induced viability, EMT, migration and sphere-forming ability of these cells. Mechanistically, CCN5-suppression by leptin is mediated via activating JAK/AKT/STAT-signaling pathways.

**Conclusions:**

These studies suggest that CCN5 serves as a gatekeeper for leptin-dependent growth and progression of luminal-type (ER-positive) BC cells. Leptin may thus need to destroy the CCN5-barrier to promote BC growth and progression via activating JAK/AKT/STAT signaling. Therefore, these observations suggest a therapeutic potency of CCN5 by restoration or treatment in obese-related luminal-type BC growth and progression.

## Background

Breast cancer (BC) is a genetically heterogeneous disease; it is the most frequently diagnosed and the second leading cause of cancer-related death in women in the United States and globally [1–3]. It attacks one in eight women (~ 12%), impacting nearly every family worldwide [4–7]. In both pre- and post-menopausal women, one of the important risk factors for BC is obesity [8–11], which is associated with increased risk of recurrence, resistance to chemotherapy, poorer survival and overall adverse disease prognosis [12–14]. The mechanisms through which obesity may influence the disease process include an excess production of estrogen by adipose tissue aromatase (peripheral aromatization), reduced levels of sex hormone-binding globulin with consequent rise of the bioactive/free estradiol, increased biosynthesis of insulin-like growth factors (IGFs) and adipose tissue secreted factors like leptin, which is involved in various physiological functions such as sense of satiety, energy metabolism, fertility, immune response and hematopoiesis [15, 16]. The action of leptin is mediated via its receptor (Ob-R) that in turn can stimulate the signaling pathways like Jak/Stat3, ERK1/2, and PI3 Kinase/Akt [17–19]. Additionally, leptin can crosstalk with other signaling systems in BC cells [20, 21]. The studies have shown higher serum levels of leptin in patients with BC [22–25]. Furthermore, leptin over-expression in BC has been found to be associated with more aggressive clinical features [26–28]. Several investigators observed a stimulating effect of leptin on aromatase activity [29–31], and activation of ERα in BC cells [32]. However, in contrast, the elevated levels of leptin may provide resistance to anti-estrogen therapy in BC patients [33]. The leptin signaling may promote abnormal angiogenesis and permeability as leptin has been shown to stimulate the expression of vascular endothelial growth factor (VEGF) and its receptor [34]. Furthermore, the invasive properties of BC cells have been shown to augment by leptin through a novel bidirectional crosstalk between leptin and IGF-I signaling that could transactivate epidermal growth factor receptor (EGFR), an important member of HER2/neu family [35]. A robust influence of leptin on extracellular matrix (ECM) has been demonstrated [36]. It is known that different components of ECM constitute the tumor microenvironments that significantly affect the pathological process of tumor invasion as well as progression. Thereby, leptin-mediated regulation of ECM proteins may help in promoting invasion and metastasis in BC.

A group of ECM-associated cysteine-rich proteins that belong to the CCN (Cyr61, CTGF, -Nov) family of growth factor have recently emerged as multifunctional molecules, which modulate various cellular functions [37–39]. CCN5 (WISP-2) is a multi-modular-matricellular protein (~ 29–35 kDa) with a long half-life, and a member of the CCN family [38, 40–42]. The transient expression of CCN5 has been detected in fetal lung, adult skeletal muscle, colon, ovary, and breast [38, 43, 44]. CCN5 has been implicated as having an important role in carcinogenesis, with particular relevance to human breast disease [38, 41, 45–48]. In most studies, CCN5 expression has been shown to correlate inversely with the aggressiveness of cancers in breast [38, 49, 50], pancreas [51, 52], salivary gland [53], gallbladder [54] and gastric tissue [55], suggesting tumor suppressor/anti-invasive activity [38, 41, 52]. Thus, at least in BC, CCN5 can be considered a good prognostic marker [44]. Multiple studies have shown that CCN5-overexpressed BC cells are less aggressive in nature compared to CCN5-under expressed or negative BC cells [38]. CCN5 expressing BC cells (e.g. MCF-7, BT-474, ZR-75-1, T-47D) are always ER-α positive (luminal type), while CCN5-negative cells are mostly triple-negative (ER-, PR- HER2-)-breast cancer (TNBC) cells (e.g. MDA-MB-231, MDA-MB-468, HCC-70, BT-20, MVT-1 and 4 T1) [38, 46, 52], which are enriched with tumor initiating cells (TICs)/cancer stem cells. Mechanistically, multiple genetic insults, including the gain of p53 mutations, deplete CCN5 expression at the transcription level in noninvasive BC cells and help cells gain invasive phenotypes [56]. Moreover, several oncogenic lesions such as miR-10b upregulation and activation of TGF-β-signaling can accumulate during CCN5 crisis in BC cells [38, 48, 57].

CCN5 regulation in ER-α-positive cells is estrogen, an insulin-like growth factor and HIF-α2-dependent, and its expression has been found to participate in controlling proliferation as well as the aggressive phenotypes of these cells [38, 44, 50]. Thereby, CCN5 depletion in ER-α-positive cells promotes estrogen-independent growth, epithelial-mesenchymal transition (EMT), and stemness, consistent with more invasive phenotypes and display similarities to TNBC. In contrast, ectopic expression of CCN5 in TNBC cells reduced growth/proliferation, tumor-forming ability, invasiveness and sensitivity to tamoxifen by activating ER-α, demonstrating similarities to ER-α positive, non-aggressive BC cells [44, 47, 58]. Thus, these studies reveal that the CCN5-signaling could be a driving force to prevent TNBC growth and aggressiveness [44].

Given the tumor suppressor and anti-invasive roles of CCN5 in BC, we used molecular techniques to investigate whether leptin has any influence on CCN5 to promote BC progression. We found that leptin suppresses CCN5 in BC cells to promote its pathobiological functions. The suppression of CCN5 is mediated through JAK/STAT3-Akt signaling pathway.

## Methods

### Reagents and antibodies

Dulbecco’s modified Eagle’s medium (DMEM), penicillin, streptomycin, Aprotinin, PMSF, Leupeptin, trypsin EDTA solution, sodium pyruvate, 17β-estradiol (E2), leptin and β-actin monoclonal antibodies were purchased from Sigma Chemical Co. (St. Louis, MO, USA). Human recombinant CCN5 protein (hrCCN5) was purchased from PeproTech (Rocky Hill, NJ, USA). Anti- E-cadherin and anti-vimentin antibodies were purchased from BD Biosciences (Franklin Lakes, NJ, USA) and Thermo Fisher Scientific (Waltham, MA, USA), respectively. Anti-WISP-2/CCN5 rabbit polyclonal antibody and Anti-Snail antibody were purchased from Abcam (Cambridge, MA, USA). Super Signal ULTRA chemiluminescent substrates were obtained from Pierce, Rockford, IL. Cell-death detection ELISA kits were purchased from Roche Diagnostic (Indianapolis, IN). The authentication certificates for all these chemicals, drugs and antibodies were provided by these companies. The fresh working solutions of the chemicals and drugs were prepared once a month to guarantee effectivity.

### Cell lines and cell culture

The estrogen receptor-α (ER-α) positive MCF-7, ZR-75-1 cell lines and MDA-MB-231 triple negative breast cancer (TNBC) cell lines were purchased from American Type Culture Collections (ATCC, Manassas, VA) and grown in Dulbecco’s modified Eagle’s medium (Sigma Chemical Co., St. Louis) supplemented with 10% fetal bovine serum (FBS) (HyClone, Road Logan, UT) and antibiotics at 37 °C in a humidified chamber with 5% CO_2_. Initially, cells were grown in complete media until the culture became ~ 60–70% confluent. For all experiments unless otherwise specified, the medium was changed into serum and phenol red-free media. Then 24 h later, the medium was changed into serum and phenol red-free media, and the cells were treated with Leptin or hrCCN5 or combination as per the requirements of the experiments.

### Cell viability assay

Cell viability assay was performed as described earlier [59, 60]. Briefly, MCF-7, ZR-75-1 and MDA-MB-231 were plated with 1 X 10^4^ live cells per well in 96-well culture plate. Plates were maintained at 37 °C in a humidified atmosphere with 5% CO_2_. About 60–70% confluent cells were serum-starved for 24 h to synchronized them. Cells were then treated with different doses of leptin or hrCCN5 protein (10.29 nM) or both for different time points in serum free DMEM as needed for the experiments. Cells were stained with crystal violet solution for 10 min. Cells were washed with tap water and then air dried for 30 min. Crystal violet stained cells were solubilized with 10% acetic acid and optical density was quantitated in Microplate reader at 600 nm. Eight wells were examined for each condition, and the experiments were repeated three times.

### Apoptosis assay

Photometric enzyme immunoassay for quantitative in vitro determination of cytoplasmic histone-associated DNA fragment after apoptotic cell death was determined as described previously [61]. Briefly, MCF-7 cells and ZR-75-1 serum starved cells were treated with leptin (3.125 nM) for 48 h. Cells were harvested and lysed with lysis buffer supplied with cell-death detection ELISA kits (Roche Diagnostic Corporation, Indianapolis, IN). The cytoplasmic supernatant was collected, and the total protein was estimated. A total of 20 μl of cell lysate containing 12-15 μg of protein was added in the streptavidin-coated microplate and allowed to react with 80 μl of buffer mixture containing anti-histone-biotin and anti-DNA-peroxidase was added and incubated on shaker under gently shaking for 2 h at 25 °C. Microplates were washed with incubation buffer for 3 times. The ABTS [2, 2′-azino-di-(3-ethyl-benzthiazoline-6-sulfonic acid)] chromogen substrate solution was added and allowed to incubate on plate shaker until the color development is sufficient for a photometric analysis (approximately 10–15 min). Color intensity was measured using ELISA plate reader (Spectramax 340, Molecular Devices) at 405 nm.

### Western blot analysis

Treated or untreated cells were washed with phosphate buffered saline (PBS) and lysed in 50 mM Tris-HCl at pH 7.5, 150 mM NaCl, 0.1% SDS, 1 mM PMSF, 1 ng/ml leupeptin and 1 ng/ml Aprotonin or phospho-lysis buffer, sonicated for 3 s and incubated on ice for 20 min. The lysates were centrifuged at 18, 000 g for 60 min at 4 °C, and the supernatants were collected and Western immunoblotting were performed as described earlier [62]. Briefly, equal amounts of proteins were resolved on 7.5% or 10% SDS-PAGE, transferred onto nitrocellulose membranes, and reacted with specific primary antibodies at 4 °C, overnight. The antigen-antibody reactions were probed with HRP-conjugated anti-rabbit or anti-mouse IgG. Immunoreactions were detected by ECL chemiluminescence reagent kit. Relative expressions of proteins were calculated by densitometric analyses using ID Image Analysis Software version 3.6 (Eastman Kodak Company, Rochester, NY).

### RNA extraction and real-time RT PCR

Total RNA extraction and cDNA synthesis, were essentially the same as that previously described [63]. Briefly, total RNA was extracted from MCF-7 cell lines using TRIZOL (Invitrogen, Carlsbad, CA) as per the manufacturer’s protocol. 500 ng of total RNA was reverse transcribed using oligo d(T)_16_ primers. Real-time PCR was performed on an Applied Biosystem Step One real-time PCR system (Foster City) using SYBR Green DNA detection dye. PCR was performed for 15 s at 95 °C and 1 min at 60 °C for 40 cycles followed by the thermal denaturation protocol. C_T_ values for WISP2/CCN5 are normalized to human GAPDH by subtracting the average C_T_ value for each sample. Relative quantification (RQ) values for CCN5 mRNA in experimental samples were determined using the 2^-ΔΔCT^ method [64]. The sequences of primers are as follows: WISP-2/CCN5: 5’-CCT ACA CAC ACA GCC TAT ATC-3′ (forward) and 5’-CCT TCT CTT CAT CCT ACC-3′ (backward); GAPDH: 5′-ATG AGA AGT ATG ACA ACA GCC-3′ (forward) and 5′-TGA GTC CTT CCA CGA TAC C-3′ (backward).

### Northern blot analysis

The nonradioactive Northern blot analysis was carried out per our previous method [62]. Briefly, total RNA was separated on 1% agarose gels containing 2.2 M formaldehyde in MOPS buffer and blotted on super charged nylon membranes (Schleicher & Schuell Inc. Keene, NH). Membranes were probed with nonradioactive DIG-labeled human WISP-2/CCN5- and glyceraldehydes-3-phosphate dehydrogenase (GAPDH)-specific cDNA probes. The rest of the procedure was carried out per the protocols provided by DIG high prime DNA labeling and detection kit (Roche Diagnostics GmbH, Indianapolis, IN). The signal intensities of WISP-2/CCN5 and GAPDH were measured by densitometric analysis using one-dimensional image analysis software (Kodak Image Station, version 3.6) for normalization.

### Transwell cell migration assay

Cell migration assay was performed per the method described by Maity et al. [59]. Briefly, semiconfluent MCF-7 and ZR-75-1 cells were serum starved for 24 h prior to the treatments and then under serum starved conditions cells were exposed to leptin (3.125 nM) or vehicle (1xPBS) in the presence or absence of hrCCN5 (10.29 nM) for 48 h. Cells (10,000 per well) were then seeded on transwell filter insert of 8-μm pore size (Becton Dickinson) coated with fibronectin (10 μg/ml). DMEM with no serum was added into the upper wells while DMEM with 10% FBS were added into the bottom chamber. The cells were incubated overnight for migration towards serum at 37 °C with 5% CO_2_. Cells adherent to the upper surface were removed with cotton swabs, and migratory cells attached on the undersurface were stained with crystal violet solution. Wells were gently rinsed with water and dried in the air. Crystal violet-stained attached cells were solubilized with 100 μl of 10% acetic acid and cell migration towards serum was quantitated using microplate reader at 600 nm.

### Chloramphenicol acetyltransferase assays

Chloramphenicol acetyltransferase (CAT) assay, using CAT-ELISA kits (Roche Applied Science, Inc.), was performed same as described previously [65, 66]. Briefly, CCN5/WISP-2-CAT promoter constructs (pCCN5-CAT, cloned into the pCAT-3-Basic Vector, Promega) containing the 1.9-kb human CCN5 gene promoter sequence (− 1919 to + 13) were transiently transfected into the MCF-7 cells using the Lipofectin (20 μg/ml) method [62]. After 48 h, transfected cells were grown in serum-deprived media and exposed to leptin (3.125 nM) for 48 h. Cells were harvested, and cellular extracts were prepared for CAT assays per the manufacturer’s instructions. CAT activity was measured at 405 nm using a microplate (ELISA) reader (Spectramax 340, Molecular Devices).

### Mammosphere assays

A mammosphere assay was performed as described recently [59] with little modification. Briefly, MCF-7 cells were grown in serum starved media as described earlier and treated with leptin (3.125 nM) or vehicle (1xPBS) in the presence or absence of hrCCN5 (10.29 nM) for 48 h. Cells were then seeded (0.5 cell/ml/well) into a 96-well-non-adherent micro-space cell culture plate (Elplasia, Kuraray, Co., Ltd., SQ200100NA96, Japan) containing Mammocult media (Stem Cell Technologies) with proliferation supplements, 4 μg/ml heparin and 0.48 μg/ml hydrocortisone. Single cell suspensions were allowed to grow and mammospheres were counted at day six following seeding the cells. Photographs were taken using a Leica photomicroscope, and sizes were determined using NIS-Elements software.

### Statistical analysis

The statistical analysis was performed using the Graph Pad Prism 4 software and PASS^15^ softwares. We calculated the required sample size for in vitro studies using an approximate method [67] is *n* = 3–8 cultures per groups and time point, assuming comparison-wise type I error of 5% and power of 80% to detect the probability of concordance of 75%. All data are expressed as the mean ± SEM. Statistically significant differences between groups were determined by using the nonpaired Student’s two-tailed *t*-test and ANOVA as per the requirement. A value of *P* < 0.05 was considered statistically significant.

## Results

### Leptin induces ER-α-positive BC cell viability in a dose and time dependent fashion

**Fig. 1:**
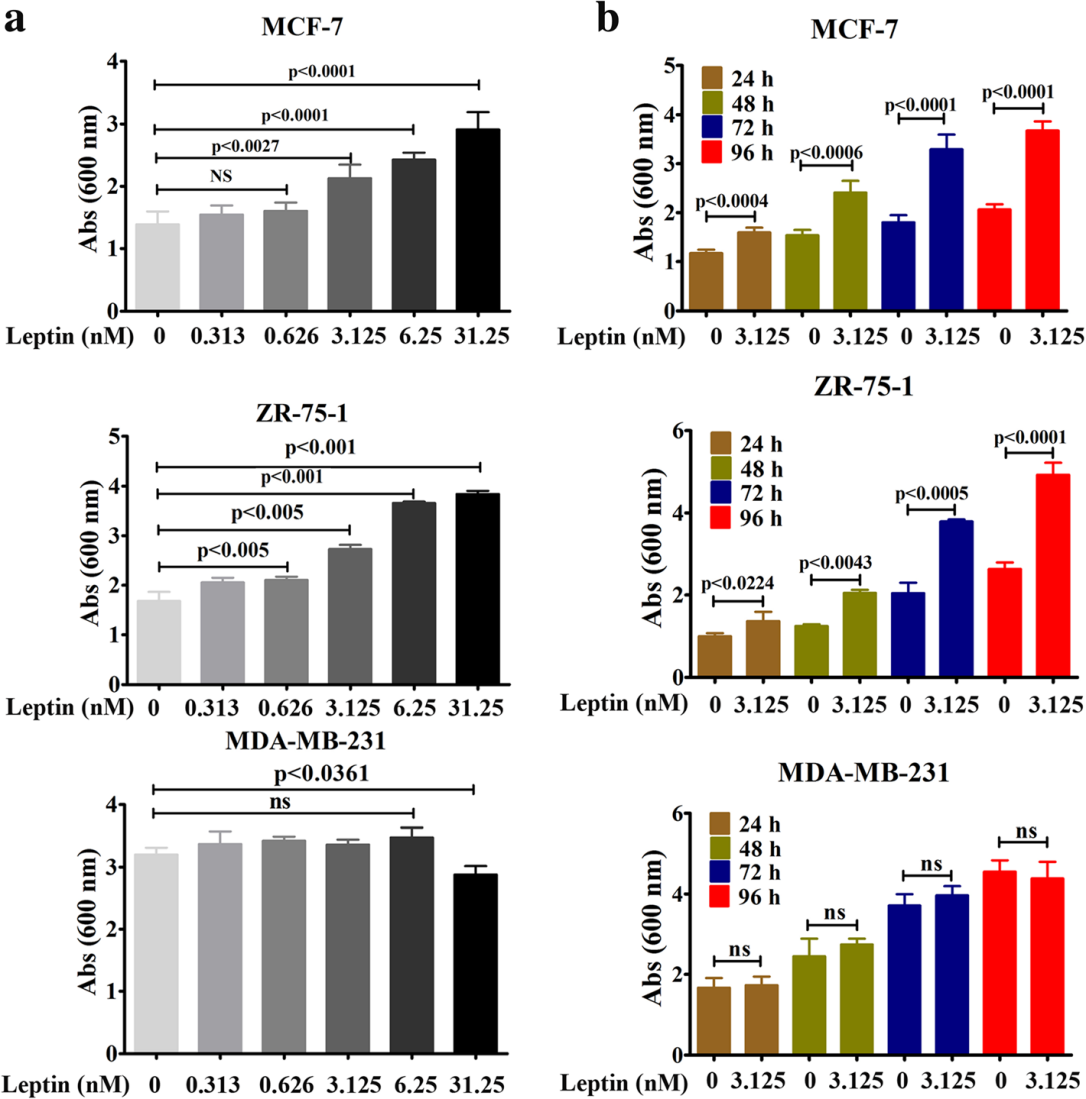
Dose- and time-dependent effect of leptin on BC cell viability. **a** Dose-Dependent effect- ~ 60–70% confluent MCF-7, ZR-75-1 and MDA-MB-231 cells were grown in serum-deprived DMEM for 96 h in the presence or absence of different doses of Leptin and cell viability was measured using Crystal Violet assay. The data represents mean ± SEM of eight independent experiments. **b** Time-dependent effect- ~ 60–70% confluent MCF-7, ZR-75-1 and MDA-MB-231 cells were grown in serum-deprived DMEM for different times (i.e., 24 h, 48 h, 72 h and 96 h) in the presence or absence of Leptin (3.125 nM) and cell viability was measured using Crystal Violet assay. The data represents mean ± SEM of eight independent experiments

Previously, it has been reported that leptin promotes MCF-7 cell growth [27, 36, 68, 69]. In this study, our goal was to investigate the effect of leptin on cell viability of different ER-α-positive and triple negative BC cell lines. To do so, ER-α positive cells (MCF-7 and ZR-75) and TNBC cells (MDA-MB-231) were serum-starved for 24 h to synchronized the cells and then treated with different doses of leptin (i.e. 0, 0.313, 0.626, 3.125, 6.25, 31.25 nM) for 96 h or single dose (3.125 nM) of leptin for different time points (i.e., 24 h, 48 h, 72 h, and 96 h). Following treatments, cell viability was measured using Crystal Violet Assay. As shown in Fig. 1, leptin treatment significantly promotes cell viability in MCF-7 and ZR-75-1 cells in a dose and time-dependent fashion. The significant induction was first detected at 24 h with a dose of 3.125 nM. The effect was gradually increased with the increments of times and doses of leptin (Fig. 1a).

Except high dose (31.25 nM), the leptin effect on cell viability was undetected in MDA-MB-231 cells even after the treatment of 96 h. We found that 31.25 nM dose of leptin treatment minimally but significantly reduced the viability of MDA-MB-231 cells while the effect was converse in MCF-7 and ZR-75-1 BC cell lines (Fig. 1a, bottom panel). Thus, this finding suggests that higher doses of leptin may act through a complex mechanism and cellular context dependent, which has not yet been elucidated.

### Leptin suppresses CCN5 expression at the transcriptional level in ER-α-positive breast cancer cells

**Fig. 2:**
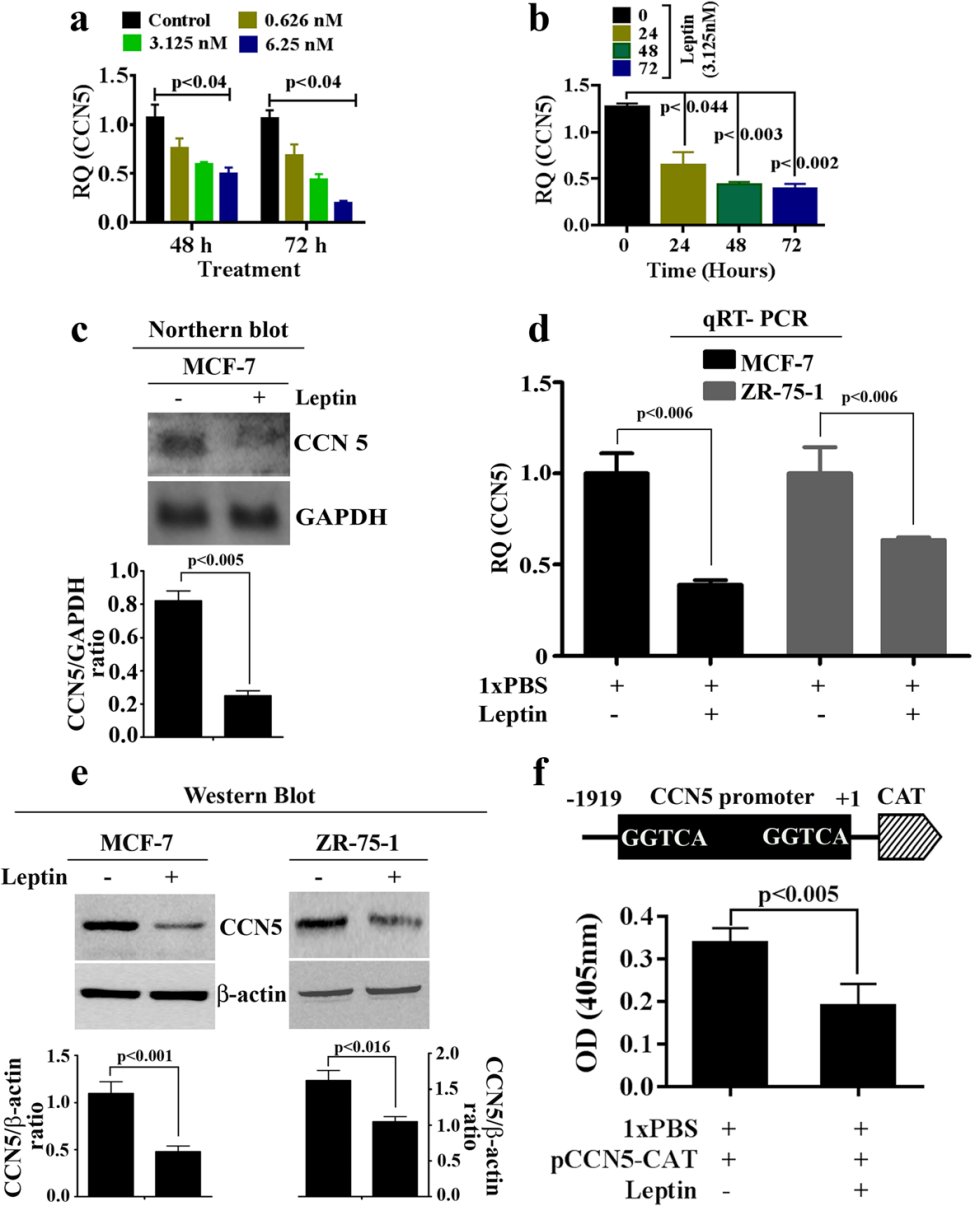
CCN5 regulation by leptin in BC cells. **a**-**b** ~ 60–70% confluent MCF-7 cells were serum deprived for 24 h and then cells were treated with different doses of leptin or different times with a fixed dose of leptin (3.125 nM). Total RNAs from treated and untreated cells were extracted and were subjected to qRT-PCR. Values on the barograph represent CCN5 expression changes in treated and untreated groups. The data represents mean ± SEM of three independent experiments. **c** Serum deprived MCF-7 cells were grown in serum-deprived DMEM for 48 h in the presence or absence of Leptin (3.125 nM), and total RNAs from treated and untreated cells were extracted and were subjected to Northern blot analysis for CCN5 and GAPDH (loading control). Values on the barograph represent CCN5 expression changes in treated and untreated groups. The data represents mean ± SEM of three independent experiments. **d** Serum deprived MCF-7 and ZR-75-1 cells were treated with leptin for 48 h as indicated above, and total RNA extracts were subjected to qRT-PCR analysis for CCN5. Values on the bargraph represent CCN5 expression changes in treated and untreated groups. The data represents mean ± SEM of three independent experiments. **e** MCF-7 and ZR-75-1 cells treated with leptin (3.125 nM) for 48 h, and whole cell extracts were subjected to immunoblot analysis for CCN5 and β-actin (loading control). Values on the bargraph represent CCN5 expression changes in treated and untreated groups. The data represents mean ± SEM of three independent experiments. **f** MCF-7 cells were transiently transfected with CCN5/WISP-2 promoter. After 48 h, transfected cells were grown in treated with 3.125 nM leptin for 48 h or left untreated, and CAT assay was performed per the protocols indicated in Materials and Methods section. The results reflect the mean ± SEM of 3 independent experiments

Recently, we found that CCN5-signaling is the driving force to prevent the growth and aggressive behavior of BC cells [44]. Thus, the goal of this study was to examine the effect of leptin on CCN5 expression in BC cells using qRT-PCR analysis. We found that the mRNA expression of CCN5 was significantly decreased in a dose- and time-dependent fashion in leptin-treated ER-α and CCN5-positive MCF-7 cells as compared to vehicle treated cells (Fig. 2a and b). As expected, the effect of leptin with different doses was undetected in CCN5-negative MDA-MB-231 cells (data not shown). To validate the above data, we further evaluated the effect of leptin on CCN5 mRNA and protein levels in MCF-7 and ZR-75-1 cells. To do so, Cells were grown to 60–70% confluences, serum starved for 24 h and then treated with leptin (3.125 nM) or vehicle (PBS, controls) for 24 h. CCN5 mRNA levels were determined using Northern blotting and qRT-PCR, and CCN5 protein level was measured using Western blot analysis. Consistent with previous data, we found that both mRNA (Fig. 2c and d) and protein levels (Fig. 2e) of CCN5 were significantly decreased by leptin treatment**.** Based on the dose-dependent effects of leptin on cell viability and CCN5 expression in MCF-7 and ZR-75-1 cells, 3.125 nM (50 ng/ml) was considered for rest of the experiments.

Since both mRNA and protein expressions of CCN5 were affected by leptin, we determined whether leptin-induced downregulation of CCN5 is mediated at the transcriptional level via binding to CCN5 promoter. To do so, CCN5-CAT promoter constructs containing the 1.9-kb human CCN5 gene promoter sequence were transiently transfected into the MCF-7 cells using the Lipofectin (20 μg/ml) method [62]. The transfected cells were then exposed to leptin (3.125 nM) or 1xPBS alone for 48 h. Cells were harvested, and cellular extracts were prepared for CAT assays. We found that CAT activity significantly impaired by leptin treatment as compared to PBS-treated samples (Fig. 2f).

Taken together, these results indicate that leptin suppresses CCN5 expression in ER-α-positive breast cancer cells at the transcription level.

### Leptin-induced viability is impaired by CCN5 treatment

**Fig. 3:**
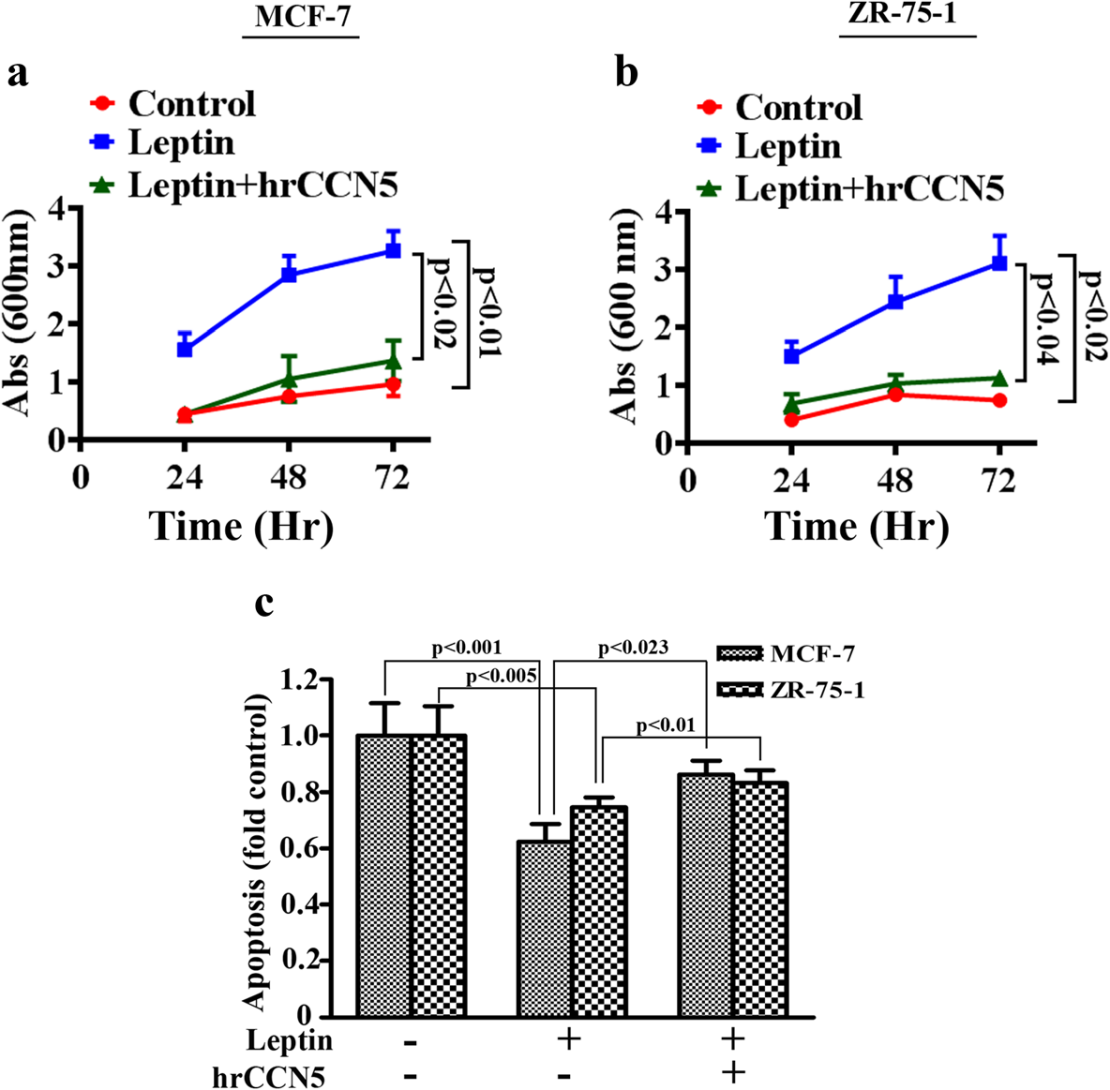
Regulation of cell viability and apoptosis by leptin is mediated by CCN5. **a**-**b** BC Cells were grown for 24 h in a 96- well plate under serum free condition. Cells were then treated with leptin (3.125 nM) or in combination of hrCCN5 (10.29 nM) in serum free media for 24 and 48 h. Cell viability was measured using crystal violet staining assay. Values on the bargraph represent the cell viability in treated and untreated groups. The data represents mean ± SEM of three independent experiments. **c** MCF-7 cells were serum deprived for 24 h and then treated with leptin (3.125 nM) in the presence or absence of hrCCN5 (10.29 nM) for 48 h under serum deprived conditions. Apoptotic cell death was determined using cell-death detection ELISA kit (detailed explanation in text). Values on the bargraph represent the apoptosis in treated and untreated groups. The data represents mean ± SEM of eight independent experiments

We next determined whether suppression of CCN5 by leptin is a relevant episode in leptin-mediated ER-α-positive breast cancer cell viability. MCF-7 and ZR-75-1 cells were first serum-starved for 24 h to synchronize cells, and then treated with leptin (3.125 nM) or PBS for different time points in the presence or absence of hrCCN5 protein (10.29 nM). The cell viability was assayed by crystal violet staining. Consistent with previous findings (Fig. 1), we found a time-dependent stimulatory effect of leptin on the cell viability of MCF-7 cells and ZR-75-1 cells (Fig. 3a and b), and the effect was significantly impaired when cells were concomitantly treated with leptin and hrCCN5 protein. Collectively, these studies indicate that leptin-induced ER-α-positive breast cancer cell viability is mediated via suppressing CCN5 expression.

### Leptin suppresses apoptotic process to promote cell viability and this episode can be blocked by CCN5

To study whether stimulation of cell viability by leptin is due to suppression of apoptosis, we performed ELISA-based apoptosis assay. Consistent with previous works, we found that 48 h leptin treatment suppressed the regular apoptosis occurred during in vitro cultured and suggesting that this episode may promote cell viability (Fig. 3c). Since the leptin effect on inhibition of apoptosis in MCF-7 and ZR-75-1 cells were not so dramatic as compared to the leptin effect on cell viability, we anticipate that other cell physiological factors could be linked with leptin-induced cell viability. Furthermore, we found that hrCCN5 protein treatment significantly rescues cells from leptin-induced suppression of apoptosis (Fig. 3c).

### CCN5 protein treatment reprograms leptin-induced invasive phenotypes

**Fig. 4:**
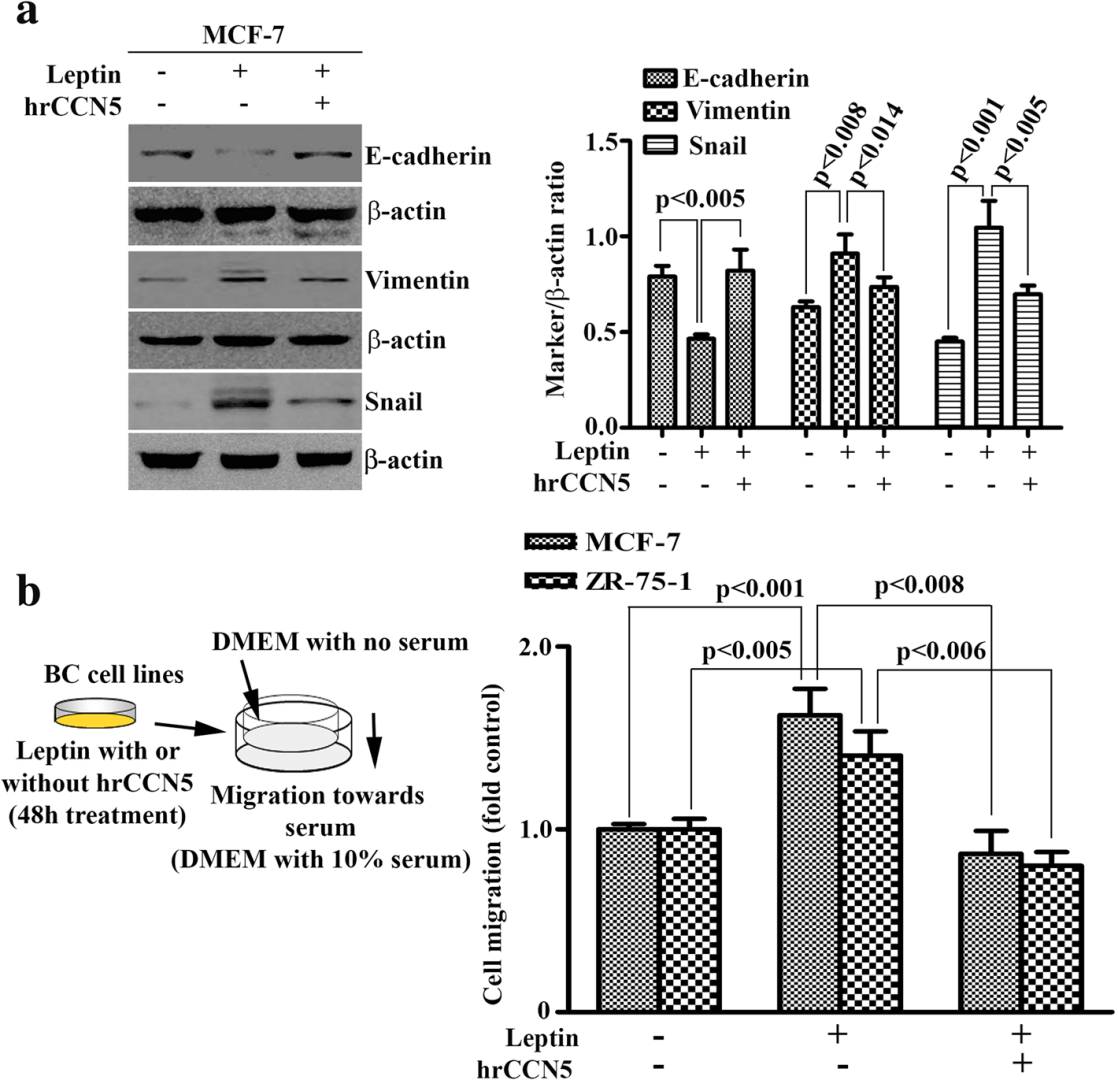
hrCCN5 protein reprograms leptin-induced epithelial to mesenchymal transition and migratory behavior. **a** Equal amount of protein lysates of MCF-7 cells treated with leptin in the presence or absence of hrCCN5 was loaded on 7.5–10% SDS-PAGE for the detection of EMT markers. A right panel shows the error bars which indicate mean ± SEM, and represents at least three independent experiments. **b** A diagram depicting experimental design to determine the leptin effect on BC cell migration in the presence or absence of hrCCN5 (left panel). MCF-7 and ZR-75-1 cells were treated with leptin in the presence or absence of hrCCN5 for 48 h and then seeded on the transwell filter insert of the modified Boyden chambers. Next day, the migrated cells were stained with crystal violet and quantitated on a microplate reader at 600 nm (right panel). The result is a representative of three independent experiments and displayed as mean ± SEM

EMT is physiological and pathophysiological events in which epithelial cells are converted into mesenchymal cells for functional needs. In cancer, EMT contributes in early stage dissemination of cancer cells followed by invasion and metastasis [70, 71] as well as drug resistance [72]. The EMT process can be induced by leptin in BC cells [21]. Since CCN5 ablation promotes EMT and invasion in BC cells [38, 47, 51, 73, 74], we sought to determine whether leptin induces EMT via suppressing CCN5 in BC cells. As expected, exposure of MCF-7 cells to leptin resulted in up-regulation of mesenchymal marker (vimentin and snail) expressions accompanied by a marked decrease in E-cadherin (epithelial marker) (Fig. 4a and b). On the other hand, leptin-induced regulation of EMT markers can be impaired by concomitant treatment of hrCCN5 protein.

Next, we investigated whether these molecular changes affect the migratory behavior of breast cancer cells. To do so, MCF-7 and ZR-75-1 cells were treated with leptin or 1xPBS in the presence or absence of hrCCN5 for 48 h under appropriate experimental conditions (see Materials and Methods section). Cells were then seeded on the upper chamber of a Boyden chamber containing serum free DMEM, and after 24 h, the migration of these cells to wards serum, which was added into the bottom chamber with DMEM, was determined. We found that the in vitro migration was significantly increased in leptin-treated MCF-7 and ZR-75-1 cells as compared to PBS-treated cells (Fig. 4b). However, leptin-induced migration was decreased to the basal level in the cells that were pre-exposed to hrCCN5 protein (Fig. 4b).

Taken together, these results indicate that leptin promotes EMT and migration of BC cells and that can be blocked by the treatment of CCN5 protein, suggesting CCN5 restoration could be beneficial for reversal of EMT via reprograming of gene-signatures.

### Leptin-induced mammosphere growth is nullified by CCN5 treatment

**Fig. 5:**
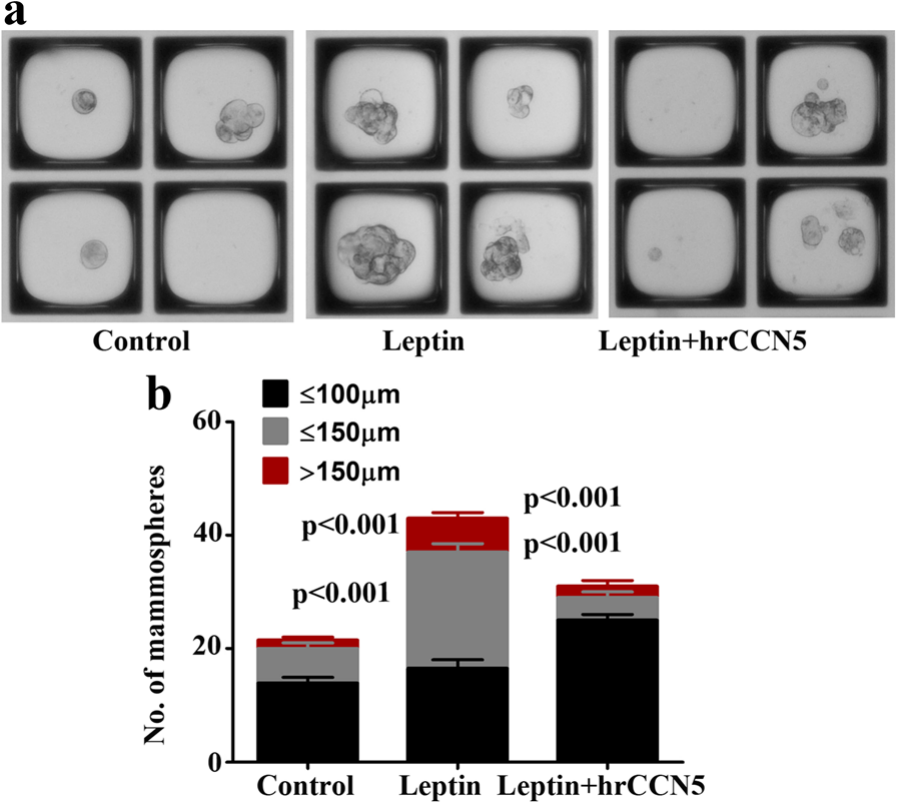
hrCCN5 treatment suppresses the mammosphere forming ability of leptin treated MCF-7 cells. **a** Representative images of MCF-7-mammospheres following leptin (3.125 nM) treatment in the presence or absence of hrCCN5 (10.29 nM). **b** Bar graph represents the number of mammospheres of different sizes in the experimental set-up indicated. Error bars indicate mean ± SEM of three independent experiments

Previous studies have shown that leptin enhances mammosphere forming ability of MCF-7 cells, and thus, suggested leptin helps in augmenting the cancer stem cell (CSCs)/tumor initiating cells (TICs) properties in less aggressive BC cells [21, 75]. We, therefore, determined whether CCN5-treatment blocks leptin-induced CSCs/TICs properties in BC cells. Consistent with previous work, we found that leptin treatment significantly increased the number and sizes of mammosphere as compared to controls (Fig. 5). In contrast, CCN5 treatment significantly blocks the leptin-induced mammosphere formation by MCF-7 cells (Fig. 5). These data suggest that CCN5 ablation by leptin is a critical pathway to induce mammosphere formation by Non-aggressive and ER-positive breast cancer cells.

### CCN5 suppression by leptin is mediated by JAK/Akt/STAT3-pathway

**Fig. 6:**
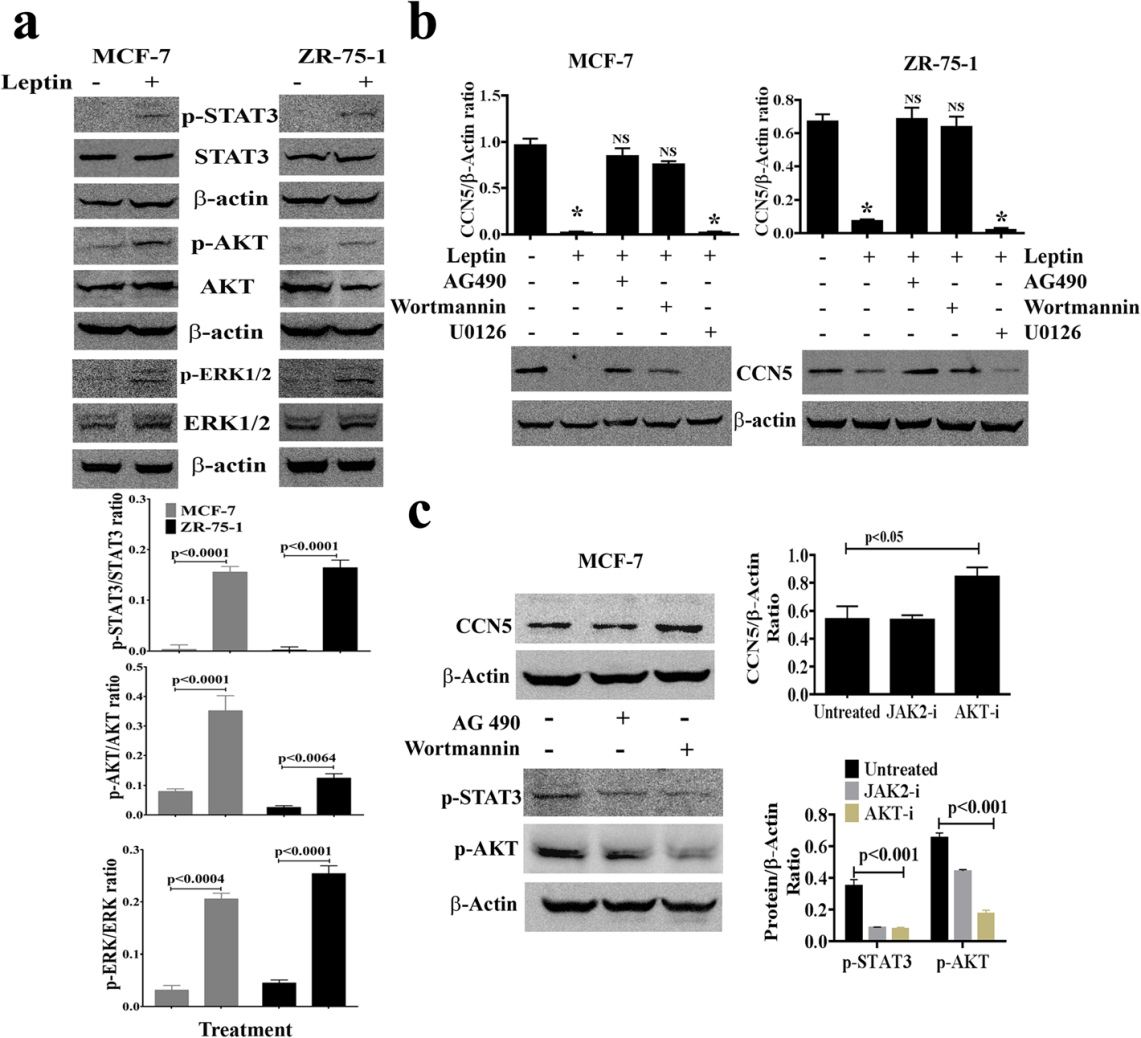
Leptin promotes CCN5 expression via activation of JAK/STAT3/Akt signaling mechanism. **a** MCF-7 and ZR-75-1 cells were serum deprived for 24 h and then grown again in serum-deprived MDEM in the presence or absence of leptin (3.215 nM) for 48 h and the status of phosphorylation of STAT3, p-AKT and p-ERK1/2 and constitutive expressions of these three proteins were measured using Western blot analysis. β-actin was used as loading controls. Error bars indicate mean ± SEM of three independent experiments. **b** Semi-confluent (~ 60–70%) MCF-7 and ZR-75-1 cells were grown in serum-deprived MDEM for 24 h, and then treated with different pharmacological inhibitors [AG-490 (100 μM), Wortmannin (20 μM) and U0126 (10 μM)] for 1 h. Following treatments of inhibitors, cells were grown in the presence or absence of leptin for 48 h. CCN5 levels were measured in the cell extracts using Western blot analysis. The doses of the inhibitors are obtained from the vendors’ instruction manuals. Error bars indicate mean ± SEM of three independent experiments. NS, non-significant, *p < 0.0001vs control. **c** Effects of different inhibitors on CCN5 expression and activities of p-STAT3 and p-AKT in MCF-7 cells. Error bars indicate mean ± SEM of three independent experiments. JAK2-i, JAK2-inhibitor and AKT-i, AKT-inhibitor

Leptin-induced growth and progression of BC cells are mediated via its receptor (Ob-R) that in turn can stimulate the signaling pathways like JAK/Stat3, ERK1/2, and PI3 Kinase/Akt [17–19, 21]. In this study, we sought to determine whether any of the above signaling pathways are involved in CCN5 suppression by leptin in BC cells. We first analyzed the effect of leptin on different signaling proteins including p-STAT3, p-AKT and p-ERK1/2 using Western blot analysis. Consistent with previous findings [17–19, 21], we found that the activities of all three signaling molecules were increased following leptin treatment in MCF-7 and ZR-75-1 cell lines (Fig. 6a). Next, we determined whether leptin-induced activation of these signaling mechanisms are linked with CCN5 suppression. To do so, we treated MCF-7 and ZR-75-1 cells with leptin in the presence or absence of pharmacological inhibitors of JAK2 (AG490), extracellular signal-regulated kinase (ERK) (U0126), or PI 3-kinase/Akt (Wortmannin). We found that blocking JAK2 and AKT activities by inhibitors significantly impaired the inhibitory action of leptin on CCN5 expression in MCF-7 and ZR-75-1 cells (Fig. 6b). However, ERK-inhibitor was unable to rescue CCN5 from leptin-induced suppression in these cells (Fig. 6b, last lane). These inhibitors alone, except AKT-inhibitor, have no significant effects on CCN5 expression in these cell lines (Fig. 6c). AKT-inhibitor induces CCN5 expression minimally but significantly in MCF-7 cells. Finally, to uncover the descending pathways, we determined the effects of JAK-inhibitor on Akt-activity and other way around. We found that JAK-inhibitor significantly decreased Akt-and STAT3 activities. Similarly, AKT-inhibitor significantly diminished STAT3 as well as AKT activities in MCF-7 cells (Fig. 6c). However, consistent with previous work [76], STAT3 inhibitor (Niclosamide) does not impair AKT signaling in MCF-7 cells (data not shown). Therefore, these studies indicate that leptin blocks CCN5 expression via JAK/ Akt /STAT3 pathway in BC cells.

## Discussion

**Fig. 7:**
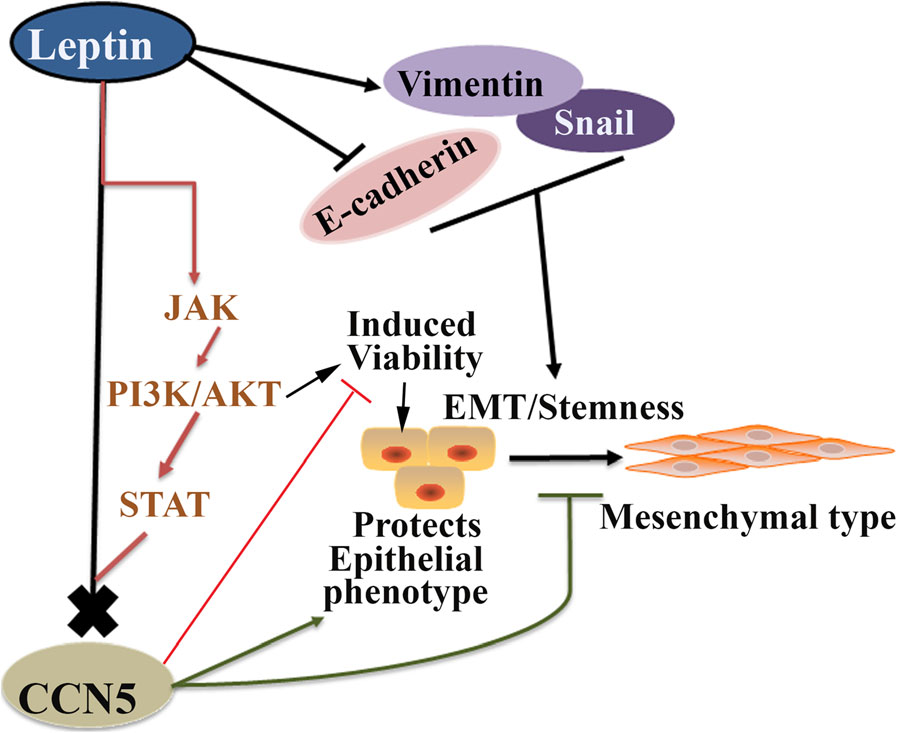
A Model depicting the role of CCN5 on leptin-induced cell viability, EMT, cell migration and Stemness. Leptin blocks CCN5 expression via activating JAK/ AKT /STAT3-signaling in luminal (ER-positive) BC cells to promote cell viability and aggressive phenotypes of these cells

Obesity is an established risk factor for BC in postmenopausal women. Current hypotheses suggest that leptin, which is also known as an obesity hormone or fat hormone, plays a vital role in BC development, and high serum leptin levels are associated with an increased risk for BC [77]. Leptin appears to be a very important factor in hormonal regulation of BC growth. However, the mechanism of leptin-induced BC development is unclear. This work shows that the growth and progression of luminal type (ER-positive) BC cells by leptin is mediated through sustained CCN5 suppression via activating JAK/ Akt / STAT3-signaling pathway (Fig. 7).

BC growth, progression and metastasis rely on multiple changes in gene-signature pattern, epigenome alterations and interactions between tumor and stromal cells [78–80]. Several endogenous factors are associated with the growth and progression of BC. One of endogenous factors is leptin, which is secreted mainly from adipose tissue but also produced by other cells including cancer-associated fibroblasts and BC epithelial cells. Secreted leptin sustains short autocrine-paracrine loops and targets cancer epithelial cells to enhance their growth, motility and invasive behaviors [77]. Additionally, the secreted leptin promotes EMT, a hallmark of cancer progression, metastasis, and chemoresistance [21, 77, 81]. Thus, we can anticipate that sustained expression of leptin may promote aggressive behavior of BC cells. Mechanistically, although the multifaceted mechanisms have been proposed as driving BC growth and progression, the involvement of functionally active classical ER-α is debatable because classical ER-α expression in BC is an indicator of a good prognosis with less aggressive behaviors [82–84], where ER-α is dysfunctional in a metastatic micro-environment and is hormone resistant [85]. Thereby, we can anticipate an unhealthy cooperation between leptin and estrogen-signaling that might promote BC growth and progression with aggressive phenotypes. Given all the potential roles of leptin in BC progression, a novel mechanism of leptin is anticipated.

Multiple studies from our laboratory and others have shown that CCN5-signaling plays a vital role in orchestrating the growth and behavior of cancer cells. CCN5 acts as an anti-invasive element in cancer cells of the breast, pancreas and GI tract. [38, 41, 52, 55, 73, 74]. CCN5 is a 29–35 kDa secreted protein with long half-life (~ 53 h), and is overexpressed in preneoplastic disorders in the human breast, including atypical ductal hyperplasia (ADH) and ductal carcinoma in situ (DCIS) compared with adjacent invasive cancer cells where expression levels were undetected, minimally detected, or only sporadically detected [38]. Consistent with in vivo results, further studies have shown that CCN5 is differentially expressed in various breast tumor cell lines and its expression profile is varied depending on the microenvironment and the aggressive nature of the cells. For example, CCN5 is constitutively expressed in less aggressive human BC cells (i.e., MCF-7 and ZR-75-1), while its expression was minimally detected in the moderately aggressive BC cell line (i.e., SKBR-3) and undetected in the highly aggressive BC cell line (i.e., MDA-MB-231). Our recent studies indicate that deficiency of the CCN5-driven program in BC promotes the growth of cancer cells and EMT, while upregulation of CCN5 is linked with ER-α-activation in both normal and cancer cells of human and mouse breast [44, 58]. Given all the roles of CCN5 in prevention of BC progression, our objective was to determine the role of CCN5 in leptin-signaling networks. Consistent with previous work, leptin has been found to promote BC cell viability via suppressing apoptosis (Fig. 3); it promotes cell migration and EMT in BC cells (Fig. 4). Furthermore, leptin promotes the mammosphere-forming ability of BC cells (Fig. 5). All these pathophiological events induced by leptin can be impaired by CCN5 treatment. Thus, this study reveals a mechanism by which leptin provokes the growth and progression of BC cells through suppressing CCN5-signaling.

The above findings raised an obvious and important question as to how leptin suppresses CCN5 expression in BC cells. Previously, multiple studies have shown that leptin mainly work through JAK/ERK/AKT/STAT3 pathway [17–19, 21]. Thus, we assumed that the JAK /ERK/AKT/STAT signaling cascades could be involved in leptin-induced suppression of CCN5 expression in BC cells. Our studies support the hypothesis and indicate that CCN5 expression was suppressed by leptin via activating JAK/Akt/STAT3 pathway (Fig. 6). However, how STAT3, as a transcription factor, suppresses CCN5 in BC cells is unclear. It may directly interact with the CCN5 promoter and suppress CCN5 transcription or an intermediate pathway may involve in this event. Thus, further studies are warranted.

## Conclusions

These studies suggest that CCN5 serves as a gatekeeper for leptin-dependent growth and progression of luminal-type (ER-positive) BC cells. Leptin may thus need to destroy the CCN5-barrier to promote BC growth and progression via activating JAK/AKT/STAT signaling. Therefore, our studies uncover a novel mechanism of leptin-signaling in driving BC growth and progression and shed new lights on improving anticancer therapy.

## Abbreviations

ABTS: 2, 2‘-azino-di-(3-ethyl-benzthiazoline-6-sulfonic acid)
ADH: Atypical ductal hyperplasia
ATCC: American type culture collections
BC: Breast cancer
CAT: Chloramphenicol acetyltransferase
CCN: CTGF-Cyr61-Nov
CSC: Cancer stem cell
DCIS: Ductal carcinoma in situ
DMEM: Dulbecco’s modified Eagle’s medium
ECM: Extracellular matrix
EGFR: Epidermal growth factor receptor
EMT: Epithelial-mesenchymal transition
ER-α: Estrogen receptor-α
IGF: Insulin-like growth factor
Ob-R: Leptin receptor
PBS: Phosphate buffer saline
RQ: Relative quantification
SD: Standard deviation
TIC: Tumor initiating cells
VEGF: Vascular endothelial growth factor
